# *The Organon* and Materia Medica Club of the Bay Cities of California

**Published:** 1897-02

**Authors:** W. E. Ledyard

**Affiliations:** Secretary


### *THE ORGANON* AND MATERIA MEDICA CLUB OF THE BAY CITIES OF CALIFORNIA.

The regular semi-monthly meeting was held at the office of Drs. J. M. & C. M. Selfridge, 1068 Broadway, Oakland, Friday evening, September 18th, 1896.

The meeting was called to order by the President at 8 P. M.

Members present: Drs. J. M. Selfridge, G. J. Augur, M. F. Underwood, M. T. Wilson, W. E. Ledyard, and, as a guest, Mr. J. E. Huffman.

The minutes of the previous meeting were read, corrected, and approved.

Sections 187, 188, and 189 of *The Organon* were read and discussed as follows:

Dr. Underwood alluded to a case in which the menses had been suppressed for forty years. He wished to know if they were likely to return. During these years, after enjoying perfect health for thirty-seven years, for the last three years she has had rheumatism of a very painful character. He considered that if the menses returned the rheumatism would be cured.

Dr. Ledward mentioned a case of asthma following the suppression of itch with German (or green) soap. The soap was applied when the patient, a girl, was in her "teens;" the itch disappeared, its disappearance being followed by asthma, which, after years of treatment, continues on and off in a very modified form.

Dr. J. M. Selfridge reported the case of a lady, who, after sitting on the wet grass, had chills every other day. This was followed by heat and sweat, with thirst during all stages.

Quinine$^{200}$ was given, during two or three attacks, with no apparent effect.

Then one drop of $\theta$ in one-half a glass of water, a dose every two hours.

Then had a chill every day, alternately early and late. Gave Natrum-mur.$^{200}$ in morning after perspiration. Chills then came later. Then called Dr. McNeil, who suggested Pulsatilla, which failed to relieve.

On restudying the case he concluded Natrum-mur. to be the remedy, which was given in the CM potency, and still no relief.

Then it was noticed that there was a profuse sweat from the waist up. Calc-c.$^{200}$ was given, two doses, one hour apart.

The afternoon chill then disappeared.

On account of the sour sweat on the head Silicea$^{200}$ was given. This simply deferred the chill.

A return to Calc-c.$^{200}$ was followed by a cure.

On motion the Club adjourned, to meet again on the first Friday in October, at the office of Dr. George H. Martin, 606 Sutter Street, San Francisco.

W. E. LEDYARD, B. A., M. B.,
*Secretary.*

